# PROTOCOL: Video‐based interventions for promoting positive social behaviour in children with autism spectrum disorders: a systematic review and meta‐analysis

**DOI:** 10.1002/cl2.1171

**Published:** 2021-05-24

**Authors:** Ciara Keenan, Allen Thurston, Catherine Storey, Karolina Urbanska

**Affiliations:** ^1^ Campbell UK & Ireland, Centre for Evidence and Social Innovation Queen's University Belfast UK; ^2^ Belfast UK; ^3^ Queen's University Belfast Belfast UK

## Abstract

This is the protocol for a Campbell review. The primary objective for this review is summarising the effectiveness of video‐based interventions (VBI) in promoting prosocial behaviours in a population of young people with autism spectrum disorders (ASD). The research questions employed to fulfil this objective include: (1) Do VBI improve prosocial behaviours in children with ASD? (2) Which social skills and interactive behaviours are most successful? (3) Do VBI generally have successful rates of skill generalisation and response maintenance? (4) Do demographic characteristics (age, gender) of participants influence the effectiveness of VBI's?

## 1 BACKGROUND

### 1.1 Autism spectrum disorders (ASD)

ASD is a lifelong neurodevelopmental condition found among all races and socioeconomic groups (Wolff, 2004). ASD effects more than 1 in 100 people in the United Kingdom with diagnosis commonly taking place after the age of 3. The prevalence of ASD diagnoses is also rising, which is thought to be a result of a combination of better public awareness and improved assessment methods (Fombonne, 2005). It affects males more frequently than females with a ratio of 5:1 (Russell et al., 2014); due to the prevalence differences between males and females, there is currently a wealth of research primarily concerned with its biological origins (see Abrahams & Geschwind, 2008 for a review).

The International Classification of diseases, eleventh edition (ICD‐11, 2018) characterises ASD by repetitive and restrictive interests and behaviours, where deficits in social interaction and communication are exhibited. Due to these impairments in social interaction and communication, individuals with ASD face difficulty in expressing their wants and needs which may subsequently result in problem behaviours such as aggression and self‐injury as an alternative communication strategy (Chiang, 2008; Murphy et al., 2009).

There are a variety of diagnostic and screening tools for Autism including; The Childhood Autism Rating Scale (Schopler et al., 1980); the Pervasive Developmental Disorder Screening Test (Siegel et al., 1986); the checklist for Autism in Toddlers (Baron‐Cohen et al., 1992); the Children's Social Behaviour Questionnaire (Berument et al., 1999); the Autism Diagnostic Interview‐ Revised (ADI‐R) and the Social Responsiveness Scale (Constantino, Davis, et al., 2003). Despite their wide‐use, efforts are still being made to improve the diagnostic process for individuals displaying ASD symptoms. The ADI‐R, which is the current standard of assessment for ASD, takes over 2 h to complete which poses a threat to feasibility for use in clinical settings which are already oversubscribed with extensive waiting times for families and individuals seeking diagnosis. The Autism Screening Questionnaire has demonstrated potential in the diagnostic process, however, each of its 40 items assess either the presence or absence of a given symptom rather than using a quantitative scale to rate symptom severity therefore it has exhibited floor effects in previous research (Rutter et al., 1999). The most recent assessment tool, the Social Responsiveness Scale, adopts a quantitative approach to measuring an individual's ability to engage in appropriate social interactions using a 65‐item questionnaire. The assessment takes 15‐20 minutes to complete and relies on ratings from direct observations in natural settings, rather than reports from parents and caregivers. In a study by Constantino, Davis, et al. (2003) the SRS compared favourably with ADI‐R with the SRS offering additional advantages of increased feasibility in clinical and educational settings and large‐scale studies. In addition, the SRS offers the ability to quantify subtle differences in severity levels of ASD (Constantino et al., 2004).

#### 1.1.1 Severity levels of ASD

Table 1 indicates the DSM‐5's characterisation of 3 distinct levels of ASD severity based on social communication impairments, the intensity of restricted, repetitive patterns of behaviour and the level of support which the individual will require.

**Table 1:** Distinct levels of autism spectrum disorders severity based on social communication impairments

Severity level	Social communication	Restricted repetitive behaviours
Level 3 “Requires very substantial support”	Severe deficits in verbal and nonverbal social communication. Severe impairments in functioning. Severely limited initiation of social interaction. Minimal response to social cues from others.	Inflexibility of behaviour. Extreme difficulty in coping with routine/environmental change. Restricted/repetitive behaviours substantially interfering with daily functioning. Great distress and/or difficulty changing focus or action.
Level 2 “Requires substantial support”	Marked deficits in verbal and nonverbal social communication. Social impairments apparent with supports in place. Limited initiation of social interactions. Reduced or abnormal responses to social cues from others.	Inflexibility of behaviour. Difficulty coping with change. Restricted/repetitive behaviours obvious to an observer, interfering with daily functioning. Distress and/or difficulty changing focus or action.
Level 1 “Requires support”	Without supports, deficits in social communication cause noticeable impairments. Difficulty initiating social interaction. Examples of atypical or unsuccessful responses to social cues from others. Decreased interest in social interactions.	Inflexibility of behaviour causes significant interference with functioning in one or more contexts. Difficulty transitioning between activities. Problems with organisation and/or planning which reduces independence.

*Source*: Adapted from DSM 5; American Psychiatric Association (APA) (2013).

### 1.2 Social skills

Social deficits relating to ASD have long been accepted as the most difficult, debilitating and pervasive aspects of the disorder (Kanner, 1943). Deficits in social behaviour cause an array of problems in interpersonal interactions. These problems, when left to persist, affect the individual's quality of life across academic, personal relationships, community placing and vocational contexts (Gresham, 1986). Impairments in social skills may cause problems across the following areas;

#### 1.2.1 Interactive social engagement

Difficulty with instigating friendship is said to derive from the inability to share common experiences with peers and the difficulties a person with ASD has in expressing empathy or showing any awareness of another person's thoughts or feelings (Weiss & Harris, 2001); these complications with friendship experience can also become apparent in a further three ways. First, the person with ASD may try to interact but do so in an odd and socially unacceptable way (Dahlgren & Christopher, 1989). Second, the individual might choose to be isolated due to their own personal preference (Mundy et al., 1994). Finally, the TD peer may avoid the individual with ASD due to prolonged conversations about a fixed and repetitive subject that may only interest the person with ASD (Gutstein & Whitney, 2002). In addition, many individuals with ASD show minimal or no interest in social interactions (Reichow et al., 2012).

#### 1.2.2 Social understanding

Individuals with ASD often display an inability to behave in a way that displays a natural affective interchange from the presented social cue and difficulties in matching social behaviour to that of the response of a TD peer can lead to a disjointed and awkward social exchange. Individuals with ASD can often misunderstand humour, either by laughing inappropriately or ignoring attempts at humour from TD peers which can subsequently result in them becoming the object of insult or mockery (Samson & Hegenloh, 2010).

#### 1.2.3 Safety skills

The lack of social skills may also impair the acquirement of the safety skills among the ASD individuals. These skills include an avoidance or prevention of potentially dangerous situations occurring to maintain the safety of a person. Thus, special interventions need to be developed to bridge the difficulties faced by those individuals. Examples of the safety skills include pedestrian skills (Steinborn & Knapp, 1982), first aid (Gast et al., 1992), emergency use of a phone (Koegel, 1988), fire safety (Self et al., 2007) and their response to the lures of strangers (Akmanoglu & Tekin‐Iftar, 2011).

#### 1.2.4 Imaginative play

Children with ASD are described as having problems with imaginative play such as role playing characters from story books or creating their own fictional scenarios. Engaging in such play has an important function in the healthy development of a child (Nijhof et al., 2018). Children with autism will rarely take part in dramatic or spontaneous play but instead prefer a very focused and repetitive type of play such as methodically lining up their toys to match in size, shapes or colours (Stahmer, 1999).

#### 1.2.5 Academic performance

Upon entering into the school system, two social adjustments must be made in order to transition successfully; children with ASD have demonstrated problems in these two main areas of academic involvement. The first requires compliance to behavioural demands as set out by the teacher (Machalicek et al., 2007). The second requires complex social dynamics as witnessed during free play and often controlled by peers in the playground (Walker et al., 1998). The first of these demands is essential in creating an environment in which learning can take place, while the second is imperative in preventing disassociation from the school system (Bellini, 2006; Bellini & Akullian, 2007; Kozlowski et al., 2012; Tantam, 2000).

#### 1.2.6 Community and vocational skills

It is extremely important for individuals with ASD to develop functional skills which can be used for community and vocational involvement to promote independent living. Research has been implemented in various areas of community and vocational skills such as shopping and purchasing (Haring et al., 1987), washing machine use (Bereznak et al., 2012) and cooking (Matson et al., 2009). Jennes‐Coussens et al. (2006), highlight that acquiring competent skills in this context can improve the quality of life for individuals with ASD.

#### 1.2.7 Emotional regulation

An often‐overlooked problem in social difficulties is the way in which individuals with ASD relate to their own emotional difficulties on an introspective level, that is their way of coping with problems such as anxiety, stress and anger management. Often, the outward behaviour of the individual with ASD does not make any “sense” to the TD peer, that is, there does not seem to be a clear purpose. A TD peer may not fully understand why the individual is humming or flapping their arms continuously and often this misunderstanding will lead to complete avoidance of each other.

### 1.3 Video‐based interventions (VBI)

#### 1.3.1 Description and theoretical framework

VBI is an instructional method whereby an individual will watch a video of a model performing a skill in its entirety and then attempts to complete the skill in the same way (LeBlanc et al., 2003). VBI is particularly appealing to teachers, as in vivo modelling of a new skill can be costly, requires more time for training and implementation and can lack a systematic approach to instruction, subsequently rendering it less effective (Graetz et al., 2006). VBI has been used extensively as an approach for teaching targeted behaviours to individuals with ASD. Benefits include the cost effectiveness, enables teaching to become standardised (e.g., by removing inconsistencies of different teaching methods from teacher to teacher), and the little time required to implement the programme (Sigafoos et al., 2007).

VBI can trace its theoretical underpinnings directly to the science of ABA. ABA has consistently produced positive, valid and replicable results and has been a prominent framework in effective academic instruction for many students, not just limited to those with special educational needs. The principles and methods which underpin and define ABA have proved indispensable within the education setting whether through classroom motivation, instructional approaches, assessment and behavioural support and management (Dunlap et al., 2001). From this scientific basis, many subsets of interventions have been developed, including VBI, and when used in conjunction with the true principles of ABA, have proven to be highly effective. The principles and methods which underpin and define ABA include:

- Commitment to using reinforcement to encourage positive behaviours.
- Teaching methods which encourage higher levels of correct response.
- The use of extinction for problem behaviours.
- Focus on ensuring that newly taught behaviours be maintained and generalised to other.

ABA strongly emphasises the need for teaching based on the individual requirements of the subject. Interventions based on the principles of ABA are usually measured by direct observation from a trained behaviour analyst, this allows the analyst to apply reinforcement as soon as the desired behaviours occur. This type of direct observation from a trained professional also facilitates immediate intervention when an undesirable behaviour occurs, through environmental modification such as adaption of the reinforcement contingency or the withdrawal of an antecedent which is causing the undesired response. This continuing feedback leads to a productive environment where the individual is supported throughout the learning process, which develops greater likelihood of the behaviours being maintained and generalised. Both Banduras learning theory (Bandura, 1969) and ABA are the critical and core components of VBI which are defined and conceptually include (1) video feedback (VF); (2) video modelling (VM); (3) video self‐modelling (VSM); (4) point‐of‐view modelling (POV); (5) video prompting (VP); and (6) computer‐based video instruction (CBI) (Rayner et al., 2009) (Table 2).

**Table 2:** Types of video‐based interventions (VBI)

Type	Description	Research
Video‐Modelling (VM)	Participant watches a video demonstration of the target behaviour modelled by an adult, a peer, or a combination of both. Little research has been carried out to compare the effectiveness of these three subgroups and it is unknown which model type consistently leads to the most effective learning.	Meta‐analysis comparing the effectiveness of nine peer mediated interventions and five video modelling interventions in children with ASD, hierarchical linear models demonstrated that both peer mediated and VM were equally and significantly effective in improving the social skills in children with ASD. Authors confirm that these interventions are more effective in younger children with a negative coefficient of −0.05 for the variable of age (Wang et al., 2011).
Video Self‐Modelling (VSM)	Participant watches a video of themselves in a typical scenario and must self‐critique their behaviour (Thiemann & Goldstein, 2001)/Watches an edited video of them performing the target behaviour with the undesirable behaviours removed (McCoy & Hermansen, 2007).	Meta‐analysis on 14 studies used an improvement rate difference (IRD) effect size with 83.4% confidence intervals to test the effectiveness of VSM on 50 participants with developmental disabilities including ASD. A range of various prosocial outcomes were measured. The VSM interventions resulted in significant improvements for preschool, elementary and secondary aged children (Mason et al., 2016).
Point of View Modelling (PVM)	Here, the camera is directed to encompass the scene as the participant should see it, perhaps directed at a specific setting of interest or looking down as a set of hands are performing the desired task. Benefits of using this type of modelling include reducing irrelevant stimuli and thus optimising the ability of the person to focus on the specific task (Tetreault & Lerman, 2010).	PVM can assist in social and play skills (Hine & Wolery, 2006), self‐help skills (Norman et al., 2001), functional living (Shipley‐Benamou et al., 2002) and reducing disruptive transition behaviour (Schreibman et al., 2000).
Video Prompting (VP)	VP is usually filmed from the perspective of the spectator, presenting the learner with a subjective viewpoint. The video does not display the behaviour being modelled from beginning to end, instead, presenting the behaviour being modelled in stages usually based on a task analysis of the final skill. This facilitates the participant with the opportunity to watch and then perform each step of the task in time with the video.	A comparison study by Cannella‐Malone et al. (Cannella‐Malone et al., 2006) revealed that VP was significantly more effective in teaching daily living skills to adults with learning disabilities than VM, however, VM is much quicker and easier to administer than VP which may help to explain the popularity of this intervention.
Video Feedback (VF)	This self‐monitoring technique involves an individual performing the target skill whilst being recorded; the observed behaviour is then reviewed by a researcher. The individual is given the opportunity to watch these recorded behaviours and evaluate whether these were appropriate or inappropriate. The participant is often able to rate their behaviour while the experimenter provides direction and assistance in the subsequent modification.	Mechling (Mechling, 2005) reports that in attending to and processing their own behaviour, the participant can develop more accurate self‐perceptions and use this information to improve peer interactions and prosocial behaviours.
Computer‐Based Instruction (CBI)	Computer‐based instruction (CBI) is often used interchangeably with the terms multimedia and technology‐ based learning. CBI interactively presents the target skill being accurately performed using variety of platforms including; media, text, music, pictures and video footage (Mechling, 2005).	Research has shown that using CBI as an instructional method has positive effects on students, due to higher attention rates, recreational associations of computer use and thus, increased successful performance (Dautenhahn & Werry, 2004). A meta‐analysis of 22 studies reports on the effectiveness of CBI on children with ASD. The results show that CBI's significantly improved prosocial outcomes (*d* = 0.45 [0.08 to 0.86]) (Grynszpan et al., 2014).

### 1.4 How the intervention might work

VBI are an effective way of teaching a variety of skills through repeated exposure to a video which displays an adult, peer or the individual themselves performing the skill with a high degree of accuracy (Bellini & Akullian, 2007; Nikopoulos & Keenan, 2004). In effect, the video is used as a prompt which can then be faded over time as acquisition increases. Research has shown that VBI can be particularly useful for individuals with ASD because videos can be broken into clips which in turn breaks complex skills into smaller component skills which reduce the engagement time necessary with the video (Buggey, 2007).

In addition, VBI provides support to children with ASD who experience deficits in complex imitation skills which are necessary for observational learning to occur (Bandura, 1969). Although some rudimentary skills of imitation are a prerequisite for VBI (observing and attending to the model), imitation also requires a sophisticated understanding of who is an appropriate subject to mimic and which behaviours should be imitated (Kleeberger & Mirenda, 2010). VBI facilitates the participant with an appropriate model and desired set of behaviours, eliminating any distracting stimuli.

Moreover, VBI promotes increased skill generalisation and maintenance which is synonymous with the principles of effective instruction in ABA (Haring et al., 1987). In PVM the camera is directed at a specific setting of interest or a pair of hands performing a task therefore the individual is learning the skill from their own perspective and are less likely to attribute that skill to a person of a specific age, gender or race. This removes the risk that this target behaviour will come under the stimulus control of only the model, rather than the individual performing the skill independently.

Perhaps one of the most potent reasons why VBI may work is that it combines instruction with an already preferred activity for many individuals. Research suggests that using technology and watching videos is highly reinforcing for many children with ASD (Charlop‐Christy & Daneshvar, 2003). Instruction combined with reinforcement may increase a child's motivation to learn and perform a new skill (Hendricks et al., 2009).

### 1.5 Why it is important to do this review

Using the pearl harvesting method for searching developed by Sandieson (2006), the first author completed a scoping search in August 2020 (using adaptations of terms presented in Appendix A) in both ERIC (EBSCOhost) and PsycInfo (OVID). It was identified from this, that there are various relevant papers in this area, especially from 2013 when the use of handheld tablets rapidly increased among children. While there now exists a number of reviews and analyses, a thorough systematic search and meta‐analysis of the effectiveness of these VBI programmes is warranted as these existing reviews:

#### 1.5.1 Have not developed an advanced search strategy

In the meta‐analysis from Wang et al. (2011) they search five databases with the terms: (1) autism or autistic, (2) social or psychosocial, and (3) therapy or training or intervention or treatment. They limit their search to papers published from 1995–2008 to ensure consistency in the diagnosis of autism. This search returned 13 relevant papers, this is much less than the review team has located in our initial searches. The first published evidence of the use of a VBI as a treatment for a child with autism was reported by Steinborn and Knapp (1982). The study reported that it was possible to teach pedestrian safety skills to a child with ASD. The study used video recordings, video feedback and manipulating correct actions with a doll, to teach a 10‐year‐old child the conditions in which it was safe to cross the road. As this highlights that VBI was being employed as an intervention for autism from as early as 1982, then the authors may have missed many relevant papers which possibly could under or over value the results in a meta‐analysis.

#### 1.5.2 They focus on one type of VBI

A meta‐analysis exists which looks at the efficacy of POV modelling across 17 studies (Mason et al., 2013). While another meta‐analysis from the same author analyses results from VSM in their review of 23 research papers (Mason et al., 2016). These two types of VBI are also summarised in a paper from Bellini and Akullian (2007) where they present positive results from 23 research papers. This current review would set out to be inclusive of the core VBI types listed in the table above and so our sample will be much larger, which will present a much more inclusive and representative sample of children with ASD. Furthermore, by focusing on the multiple types of VBI which now exist, the authors will be able to draw conclusions regarding their relative effectiveness within the included sample.

#### 1.5.3 They have not been ASD specific

In a doctoral thesis from Mason (Mason, 2012), meta‐analytical techniques were utilised to test the efficacy of VBI on individuals with various disabilities including Attention deficit hyperactivity disorder, intellectual disabilities and developmental delays. As we are interested in the outcomes specifically for children with ASD we would choose to remain focused on this diagnosis so that we could provide information directly to the practitioners and policymakers interested in this area of disability. Severity of ASD will be categorised using the three levels of support from the DSM 5 and our search terms will include comorbid conditions so that any relevant sources will not be overlooked. This information will be independently extracted by the authors. Mason (Mason, 2012) also excluded any paper which was not been published in a peer review journal. This is a major limitation in a systematic search as grey literature should be uncovered and included to counteract publication bias.

#### 1.5.4 Effective intervention for social skills impairments are still unknown

As the prevalence of ASD diagnoses is increasing, it is crucial to understand the most effective strategies for promoting social skills and reducing the negative impact of the deficits associated with the ASD. Troublingly, a clinical systematic review carried out on over 100 of these utilised interventions suggested that an overwhelming number of approaches have little or no effect on the progression of the child, and many lack a solid, sound research foundation altogether (Ospina et al., 2008). There exists little agreement on one focused and optimal method of intervention and no single intervention has ever been shown to have consistent effectiveness with all individuals (K. Higgins & Boone, 1996). It has also been demonstrated that early interventions are more conducive to positive outcomes (Dawson & Osterling, 1997), making the findings of this review highly time‐sensitive. Thus, while research suggests some indicators of a potentially successful intervention, a more thorough examination of these factors is warranted.

#### 1.5.5 Scientific rigour behind VBI interventions should be fully explored

Interventions must be based on scientifically rigorous principles in order to measure their effectiveness and replicate those which are successful. For these reasons, a synthesis should be conducted on those treatments that currently exist, which show positive outcomes and that have been established as a result of a sound scientific research foundation (Simpson, 2001). While VBI has proven effective within the educational setting for typical students, it is currently positioned within the literature to hold significance and relevance as an intervention technique for ASD.

To summarise, the majority of the literature which has synthesised the empirical data in this field has been extremely useful as it describes major limitations in the body of knowledge, including little or no comparison between the differing VBI procedures, components and an overall lack of empirical data with sufficient sample size. However there still maintains confusion within the literature on a number of factors; First, who is the most effective model? McCoy and Hermansen (2007) reviewed the types and effects of different models and concluded; “The verdict on who is the most appropriate and meaningful model for which type of behaviour is still out…Based on the literature in this review, at this time, the models with the most significant impact seem to be self and peers” (p. 206); Second, there is little evidence to suggest exactly who would or would not benefit from VBI; finally, there is no available research which provides a measurement of predictions based on individual characteristics and how this may moderate success with a VBI programme. Indeed, some believe that individual characteristics such as visual processing and language skills may influence the effectiveness of such interventions (Delano, 2007). This review will seek to address these issues.

This systematic review carries particular importance and responsibility as it will be completed without bias and with a sole focus to attain answers from that research that is already available. There will be efforts made to find relationships and correlations between all available studies. This work will act as a guide by synthesising and analysing all the available data to present findings on the VBI which is most effective, in which circumstance and for whom. This will allow practitioners and policy makers understand the amendable components to take away or add to match individual differences and thus working towards a way of benefitting every individual across the spectrum.

This systematic review will provide a thorough, objective, and authoritative summary of the evidence in relation to the effectiveness of VBI. In almost four decades since the first published evidence of VBI for autism was reported (Steinborn & Knapp, 1982) a plethora of literature on the effects of VBI has amassed. However, little is known on which VBI is most effective, for whom, and why. Through exploratory moderator analysis, it is possible to synthesise data across this diverse set of interventions to ascertain the usefulness of each VBI and inform policy and practice.

## 2 OBJECTIVES

The primary objective for this review is summarising the effectiveness of VBI in promoting prosocial behaviours in a population of young people with ASD. The research questions employed to fulfil this objective include:

1. Do VBI improve prosocial behaviours in children with ASD?
2. Which social skills and interactive behaviours are most successful?
3. Do VBI generally have successful rates of skill generalisation and response maintenance?

And finally;

4. Do demographic characteristics (age, gender) of participants influence the effectiveness of VBI's?

To answer these questions most effectively, a thorough systematic review and meta‐analysis is required. The findings of the proposed study will be beneficial in clarifying the effectiveness of VBI and will provide future directions to researchers interested to know the circumstance/s in which they work best.

## 3 METHODS

### 3.1 Criteria for considering studies for this review

#### 3.1.1 Types of studies

Within the meta‐analysis, only studies which use a control group design will be eligible for inclusion. These groups (intervention and control) can be assigned randomly or nonrandomly. If nonrandom, only a rigorous matched‐comparison group design will be accepted for inclusion in the analysis. A matched group design consists of a treatment and control group that share similar baseline characteristics. This design allows greater confidence that observed group differences are due to the intervention rather than baseline differences. In a matched‐comparison design, the interventionists ensure equivalence between the two groups by collecting data on potential confounding variables at pretest. As randomised control trials are accepted as more rigorous than nonrandomised studies, the potential impact of nonrandom study design on effect sizes will be explored as part of the subgroup analyses and any significant influences will be controlled through meta‐regression. Control groups can include various types, such as; placebo, no treatment, waitlist, or usual treatment (standard care). Any study which includes one group pretest/posttest or in which a treatment group is compared to another treatment group without a control arm will not be eligible for inclusion. Results must be reported in a manner that permitted a reliable calculation of effect sizes. Finally, A person cannot serve as their own control, but instead must be compared against a group of untreated participants. A paper by Van Laarhove, Winiarski, Blood and Chan (Van Laarhoven et al., 2012) aimed to use video modelling to teach vocational skills to teenagers with ASD. Participants were assigned to receive two conditions each, one task involved receiving the VBI and the other receiving another form of instruction. Authors then collected data after each condition and list results as intervention and control, this paper will be excluded as the results compare the same ASD individual across two conditions.

Within the systematic review, we will provide descriptive tables on all other studies which match our predetermined inclusion criteria, the systematic review will provide data from studies carried out using single subject research designs, including; Multiple Baseline designs, combined designs, A‐B‐A‐B designs and alternating treatment designs, a full list of designs is available in Table 3.

**Table 3:** PICOS criteria for considering studies for this review

PICOS	Inclusion criteria
Population	Aged ≤3 and ≥18 years
	Professional diagnosis of ASD
Intervention	Video modelling (VM)
	Video self‐modelling (VSM)
	Point‐of‐view modelling (POV)
	Video prompting (VP)
	Video feedback (VF)
	Computer‐based video instruction (CBI)
Comparison	Placebo
	No treatment
	Waitlist
	Usual treatment (standard care)
	Attention/sham treatment
Outcomes	The interactive social engagement with peers
	Social understanding
	Safety skills
	Imaginative play
	Academic performance
	Community skills
	Vocational skills
	Emotional regulation
Study Designs	For meta‐analysis:
	Randomised control trials
	Quasi‐randomised control trials
	For narrative systematic review:
	Pretest–posttest designs
	Posttest only (nonequivalent groups)
	Single subject research designs (SSRD)

#### 3.1.2 Types of participants

Participants must be aged between 3 and 18 years old with a professional diagnosis of ASD.

As this review and analysis will encompass all published studies worldwide, an inclusion strategy of age 3–18 will support coverage for those studies which are carried out in an educational institution and will be inclusive of the first stage of schooling i.e. preschool stages in the United Kingdom, until the final years of education and transition into vocational skills. Countries such as Israel, America and Belgium have a higher school leaving age of 18 compared with the United Kingdom, Australia, and Italy who have a school leaving age of 16.

Although our review is not limited to those interventions carried out in an educational institution, we choose the school‐age population to encapsulate this population of young people.

If a study includes young people less than 3 or older than 18 then there will be an attempt to extract only the results associated with those in the eligible age range. If the results cannot be isolated due to either the author pooling the results or not being explicit with the age group, then authors will be contacted and this information will be requested.

Only participants who have a professional diagnosis of ASD as defined by the DSM‐5 will be included. We will also include participants with comorbid diagnosis with ASD in our initial searches to reduce the risk of overlooking any relevant sources. Information on whether the participant group was ASD‐only or ASD with comorbidities will be recorded to compare whether the effects of VBI are similar across comorbid and non‐comorbid populations.

When a study compares a group of people with ASD with a group of TD peers we use only the data from those participants who have a diagnosis of ASD. In a study by Rosen et al. (2017) a video modelling intervention to improve vocational skills was implemented in a group of ASD adolescents (*n* = 20) and a group of TD adolescents (*n* = 20). Although the authors describe the group of TD adolescents as a control group, the current researchers would not include this study in the meta‐analysis as the comparison is vulnerable to a range of uncontrolled factors which can introduce extraneous variability which may influence outcomes and validity of findings related to ASD.

#### 3.1.3 Types of interventions

Interventions which will be included in this review are the VBI types listed above, delivered to individuals with a professional diagnosis of ASD and aged 3–18 with an explicit objective of promoting prosocial behaviour/s.

Any studies which use video technology to collect and observe data will not be included, such as a randomised controlled trial (RCT) of a joint attention intervention in children with autism where a video was used to record teacher–child–mother interactions for outcome data. As it had no other part to play in the intervention, it would be excluded (Kaale et al., 2012).

Similarly, in a Quazi random control trial by Trimmer et al. (2017) 25 individuals with autism were compare with 25 matched controls on their emotional responses to a distressing video scene. As the video is not being used to teach a new social skill, but instead is being used as a tool to elicit a response, this study would also be excluded.

#### 3.1.4 Types of outcome measures

The primary outcome which encompasses the focus of this review will be any improvement in prosocial behaviours of children with ASD. These will include:

- The interactive social engagement with peers
- Social understanding
- Safety skills
- Imaginative play
- Academic performance
- Community skills
- Vocational skills
- Emotional regulation

##### 3.1.4.1 Duration of follow‐up

It is anticipated that the included interventions will report effects at multiple follow‐up periods after implementation of the intervention. In instances where this is the case, data relating to multiple points of follow‐up will be extracted in their entirety. This will allow us to conduct analysis on effect sizes related to similar time points and when outcomes are similar across various timepoints then an average effect size will be calculated to estimate effectiveness.

##### 3.1.4.2 Types of settings

Settings will not be restricted in any form and will include community‐based settings, vocational settings, educational institutions, after school facilities, summer schemes, treatment centres, clinical settings and the individual's home.

### 3.2 Search methods for identification of studies

The number of relevant articles which meet the predetermined inclusion criteria is finite and so keywords will be selected to ensure a literature search as inclusive as required, to capture the population of interest entirely, but specific enough to make the study feasible. It is expected that literature will be widely scattered due to the many differences within terminology. It is also acknowledged that there is a lack of similarity used within bibliographic databases for indexing keywords (Dixon‐Woods et al., 2006).

One search strategy which can tackle these issues and has been proven effective in locating the most relevant and inclusive keywords uses the “pearl harvesting” method (Sandieson, 2006; Sandieson et al., 2010). This method follows exact guidelines in order to find all relevant keywords that will locate relevant articles and has been used successfully in a systematic review (Waddington et al., 2014). The first author has received extensive training in the method directly from the developer and the process is outlined in a previous protocol (Keenan et al., 2016).

The full procedure for the generation of the search filters, and the final filters themselves, will be described in detail in the final review so that it can be fully and precisely replicated. A brief outline of various terms developed so far are attached in Appendix A.

#### 3.2.1 Electronic searches

All searches on electronic databases, websites, journals, conference and government proceedings will be carried out upon protocol approval.

Databases searches will include:

- Web of Science
- PubMed
- Education Resources Information Centre (ERIC)
- PsycINFO®
- Social Science Citation Index
- International Bibliography of the Social Sciences (IBSS)
- SCOPUS
- Social Science Research Network (SSRN)
- British Education Index (BEI)
- The Cochrane Central Register of Controlled Trials (CENTRAL)
- National foundation of educational research (NFER)
- Journal of Applied behaviour analysis (JABA)
- FRANCIS
- Australian Education Index
- Canadian Research Index

Initial scoping exercises have indicated that this selection is inclusive of the most relevant research within this area.

#### 3.2.2 Searching other resources

Every effort will be expended to retrieve all empirical studies that met the predetermined and explicit inclusion and exclusion criteria. This includes studies that appear in non‐published as well as published literature. Searches for unpublished studies will be carried out in:

- Google (web)
- ProQuest Dissertation and Theses (global)
- Dissertation Abstracts International
- PsycARTICLES
- PsycEXTRA
- PsycNET
- OpenGrey
- Directory of Open Access Repositories (OpenDOAR)

The grey literature search will be supplemented by searching for relevant literature in key journals, conferences, government repositories, reference lists of included studies and relevant reviews, and by contacting key researchers in the field.

When the search is complete the key journals in which the studies have been published, will be identified. The table of contents of these key journals will be hand searched to locate relevant research. Other narrative reviews related to the topic of interest will be searched for studies missed as will the reference lists of the included studies. Key authors in the area will be contacted via email and asked of any unpublished or ongoing research in the area. Government reports will be searched via the online portal such as GOV.uk for United Kingdom and the gao.gov for the US government accountability office.

Key conferences related to ASD and educational technologies include:

- Annual autism professionals conference
- Autism and mental health conference
- EdTechXEurope

The conference proceedings from 2018 until present will be hand searched to locate relevant material which has not yet been published. Authors will not restrict searches by year, geographical location, language or publication status.

### 3.3 Data collection and analysis

#### 3.3.1 Description of methods used in primary research

To describe, illustrate and exemplify the methods used in the present research, authors have identified a previously published RCTs that would meet the current inclusion criteria.

In the Isong et al. (2014) study, 80 children aged 7–7 years were researched. These children had a diagnosis of ASD and a known history of fear of attending the dentist as reported by their caregiver. Based on this outcome, this study would be coded as considering an independent life skill, a skill that is essential to successfully care for health and hygiene of teeth.

Author's randomly assigned students using a SAS generated 1:1:1:1 sequence to four distinct conditions. The first condition used VPM as the intervention (*n* = 20). Here, children with ASD watched a TD peer attend a dentist appointment to receive a basic hygiene appointment. Children watched the video as many times as they wished four weeks prior to the dental visit and again 15 minutes prior to the appointment (the frequency of observations of the video is not accounted for in the success of VBI as repeated exposure to the model is an instructional advantage of VBI over conventional teaching). The second condition facilitated the use of video goggles where the children could watch their favourite movie during the visit to the dentist (n = 20). The third condition used a combination of both techniques (*n* = 20). The final condition was the control group which attended a dental appointment without treatment (*n* = 20). As this is a multiarm study comparing three conditions and a factor design against a control group, usually reviewers would need to decide on how to deal with the variance in the shared control group (covered more in the next section). In this review, we would only be interested in condition 1 versus condition 4 (control). Condition 2 and 3 would not be considered as watching shows through the video goggles do not involve imitation behaviours. Thus, no form of active learning relevant to our review warrants these participants to be included.

The outcomes measured in this paper were varied. The primary outcome measured the participant's anxiety, the secondary measured cooperative behaviour, and the tertiary measured physiological arousal. In this review, we would only be interested in the second outcome relating to behaviour and so the other two physiological outcomes would not be extracted.

Authors measured this outcome of interest using the Venham Anxiety and Behavioural Scale (Venham et al., 1980), at two time points: once at the first visit and once again 4–6 months after the intervention. Unfortunately, measures of validity and reliability of the outcome measures were not provided so further details could not be drawn on this variable of interest.

The statistical methods used in this review included intention to treat after the control group lost one participant to follow‐up and the VPM condition lost three children to follow‐up. Analysis of variance and Fisher's exact test allowed a reliable calculation of effect size at posttest.

#### 3.3.2 Criteria for determination of independent findings

As shown, where a study reports findings from two or more different interventions, some of which are irrelevant to this review only those findings from the control groups and intervention groups which meet the eligibility criteria would be included. However, the addition of the other intervention group/s will be reported in the table presenting study characteristics.

In cases where a study has two interventions (multiarm), and both are relevant to our review we will first check whether they were measured against a common control group. If found that the same participants in the control condition provided the data for each intervention, we understand that by counting these participants twice we would increase the risk of providing an incorrect estimate of the variance for the effect size. To deal with this we will split the control group in half and continue to present each intervention separately in the final analysis.

Where the same outcome construct is measured but across multiple time domains, such as through the collection of both posttest and further follow‐up data, the main analysis will focus on synthesising the evidence relating to effect sizes at immediate posttest.

Often, authors will report data on the same participants across more than one outcome, this leads to multiple dependent effect sizes within each single study. If this occurs in more than 20 studies, Robust Variance estimation was conducted. This technique calculates the variance between effect sizes to give the variable of interest a quantifiable standard error (Hedges et al., 2010). If this occurs in less than 20 studies, authors will combine the dependent variable to produce one combined effect size per study.

Finally, in cases where study authors separate participants into subgroups relating to age, ASD diagnosis or gender and it's inappropriate to pool their data, these participants will remain independent of each other and will be treated as separate studies which each provide unique information.

#### 3.3.3 Selection of studies

Empirical evidence suggests that using two or more independent reviewers throughout the screening and data collection process reduces errors, and so using a single reviewer should be avoided (Buscemi et al., 2006). Likewise, Cochrane's minimal standards assert that two people are mandatory for screening and extracting outcome data, and highly desirable for the extraction of all other study variables. The titles and abstracts generated through the search strategy will be transported to EndNote and duplicates will be removed at this stage. These abstracts will be uploaded to the screening software Abstrackr and reviewers will be trained to screen the abstracts of located references.

Reviewers will be trained in using the online screening software and the review team will describe the objectives of the review and examples of papers which will, and will not, meet inclusion criteria. Prior to screening, the independent reviewers will also be assigned the same batch of 100 studies to establish internal consistency.

The lead review author will screen all abstracts while the trained reviewers will be assigned batches of 500 abstracts each time. Screening will be complete when all abstracts have been reviewed twice. Reviewers will make decisions to either include, query or exclude an abstract. All decisions will be checked for consensus and disagreements. Those studies in which both reviewers independently agree to include will move forward for full‐text screening, those studies which both reviewers independently agree to exclude will be removed from the library, and those studies in which reviewers query or disagree, consensus will be reached either through discussion or via the involvement of a third reviewer.

After abstract screening, remaining studies will be carried through to full‐text screening. These studies must be located and downloaded for full‐text review. The PDFs for these studies will be saved to Eppi‐Reviewer 4 (ER4) and stored under a unique study ID.

The lead review author and a trained reviewer will then use the inclusion criteria (outlined in Table 3) to independently decide whether the full‐text study should be included, queried, or excluded.

Interrater agreement will be calculated using the “irr” package in R statistical software. The Fleiss’ *κ* statistic (Fleiss, 1971) will be used to measure reliability across multiple raters at abstract screening; this approach will assess the degree of agreement across the first 100 pilot studies. The standard *κ* statistic (Cohen, 1960) will be utilised in full‐text screening stage; this approach will measure agreement on full‐text decisions between two researchers.

#### 3.3.4 Data extraction and management

Data abstraction sheets will be designed by the authors and piloted by trained research assistants using Eppi‐Reviewer. A minimum of the following data will be extracted from each included study:

- Publication details
- Geographical location of study
- Demographic variables relating to the participants including age, ethnicity and gender
- Details relating to ASD diagnosis and existing comorbidities
- Intervention details including setting, dosage and implementation
- Delivery personnel and measures of internal validity and reliability
- Descriptions of the outcomes of interest including instruments used to measure
- Design and type of trial
- Sample size of treatment and control groups
- Data required to calculate Hedge's g effect sizes
- Quality assessment

#### 3.3.5 Assessment of risk of bias in included studies

Two reviewers will independently assess the risk of bias across all included studies in the meta‐analysis using the Cochrane Risk of Bias Tool. This instrument evaluates bias as high, low or unclear across each of the following domains: Selection bias (allocation concealment and random sequence generation), performance bias (blinding of personnel and participants), detection bias (blinding of outcome assessors), attrition bias (incomplete outcome data), reporting bias (selective outcome reporting), and any other bias.

For those studies which use case series studies and pre–post designs without a control group, study quality will be assessed using tools developed by the National heart, lung and blood institute (2014).

#### 3.3.6 Measures of treatment effect

Meta‐analysis will be conducted to test effectiveness of VBI across various domains relating to prosocial behaviour. The outcomes related to social behaviour are continuous and so the effect size metric chosen is Hedges’ g, many studies will need to be recalculated into a standardised mean difference (SMD) with a 95% confidence interval to allow appropriate summary of effect sizes across the included studies.

#### 3.3.7 Unit of analysis issues

If authors report data on the same participants across more than one outcome, the meta‐analysis will use robust variance estimation to adjust for effect size dependency (Hedges et al., 2010). The correction for small samples (Tipton & Pustejovsky, 2015) will be implemented when necessary.

#### 3.3.8 Dealing with missing data

SMD will be calculated from means and standard deviations in the first instance, however, if a study does not provide this raw data, authors will be contacted and this information will be requested. Failing this, many papers have been published to assist authors in calculating the SMD from primary research (Rosnow & Rosenthal, 1996; Rosnow et al., 2000), and have enabled authors to transform many statistical tests of significance such as *t* tests, *F* tests, and *χ* ^2^ values to a metric which allows comprehension of the magnitude of the intervention effect. A very useful online calculator has also been developed, this allows authors to choose the type of raw data available, and the calculator will automatically transform this to various effect size types, including the SMD (Lipsey & Wilson, 2001).

When all methods have been exhausted, the study will be removed from meta‐analysis and reported narratively instead.

#### 3.3.9 Assessment of heterogeneity

If it transpires that there is substantial heterogeneity between studies, authors understand it is not suitable to combine these in a meta‐analysis as the experimental effects are more different than one would expect based on chance alone. Statistical heterogeneity or lack thereof will be checked in several ways. First, visually using forest plots and checking for overlap of confidence intervals. Secondly, using tests such as the Cochran *Q* test (*χ* ^2^), percentage of total variation across studies (*I* ^2^) and the *τ* ^2^ statistic (*τ* ^2^ or Tau2).

#### 3.3.10 Assessment of reporting biases

A funnel plot and Egger's linear regression test will be included to check for publication bias across included studies (Sterne & Egger, 2005). Where the funnel plot is asymmetrical this indicates either publication bias or bias which relates to smaller studies showing larger treatment effects. The trim and fill method will be used where the funnel plot is asymmetrical (J. P. T. Higgins et al., 2019), this is a nonparametric technique which removes the smaller studies causing irregularity until there is a new symmetrical pooled estimate, the studies which were eliminated where then filled back in to reflect the new estimate.

#### 3.3.11 Data synthesis

All analyses will be conducted using R software. A random‐effects analysis (REM) is chosen as the hierarchical linear model. This decision to employ a REM was made for two reasons. Firstly, it was agreed that with the type of VBI analysed the true effect would vary from study to study due to the distribution of effects. These variances may include: the setting of the intervention, the training of the person delivering the program or the dosage of the intervention. Second, under the random‐effects model the weights assigned to each individual study are more reasonable as it considers that the effect observed within each study are based on a sample from a population with an unknown mean.

#### 3.3.12 Subgroup analysis and investigation of heterogeneity

To investigate any observed heterogeneity, the following factors are some which will be explored using subgroup analysis:

1. VBI Type. The principal category of interest is the intervention type employed within the study.
2. Dosage of intervention. A distinction will be made on those interventions which have a low dosage—conducted over a shorter period (e.g., <3 weeks with a low frequency <twice per week) to those which have a high dosage.
3. Severity of ASD. This will be classified independently by the authors to categorise participants into those which require Level 1, Level 2 or Level 3 support using the recommended DSM 5 guidelines.
4. Presence of comorbidities. Inclusion of comorbidity information will ensure that any relevant sources are not missed in the search strategy. Authors will ensure that where any data relating to comorbid disorders is reported, this will be extracted in the review. This will allow us to objectively assess the impact the addition of these studies/participants will have on the final analysis.

#### 3.3.13 Sensitivity analysis

To ensure robustness of the review and to account for individual studies that appear to exert an undue influence on findings, process sensitivity analysis will also be carried out on domains relating to the quality of the included studies. The first will remove studies which were quazi‐random, leaving only RCTs. The second sensitivity analysis may leave only those studies determined as low risk overall on the Cochrane Risk of Bias assessment. The third analysis could be to remove those studies where the effect size had to be recalculated from other statistical tests, this would leave only those with effect sizes calculated directly from means and standard deviations, and this may confirm that the way missing data was dealt with was appropriate. Reporting of these sensitivity analyses will be presented in a summary table.

## AUTHOR CONTRIBUTIONS

- Content: Storey lectures on the MSc in Applied Behaviour Analysis at QUB. Storey researches specifically in the area of computer‐based instruction in improving educational outcomes and has extensive experience in clinical settings with children and adults with ASD. Keenan holds an MSc with distinction in Autism Spectrum Disorders.
- Systematic review methods: Keenan is a Research Fellow for Campbell UK & Ireland and the Associate Director of Cochrane Ireland. Keenan has an established international reputation in evidence synthesis methodology.
- Statistical analysis: Thurston has extensive knowledge in study designs and has completed many interventions in the school setting. Thurston predominantly uses randomised controlled trials (RCTs) and is currently running two‐large RCTs on improving literacy levels for students in schools. Thurston is author on a new SAGE book about trials within the educational setting.
- Information retrieval: Keenan is an information retrieval specialist for the Campbell Education Coordinating group.

## CONFLICT OF INTERESTS

The authors declare that there are no conflict of interests.

## PRELIMINARY TIMEFRAME

Approximate date for submission of the systematic review: Feb 2021.

## PLANS FOR UPDATING THIS REVIEW

Keenan will be responsible for updating the review every 3 years.

## APPENDIX A PRELIMINARY SEARCH TERMS

### Autism terms

autis* OR "pervasive developmental disorder*" OR "pervasive developmental delay*" OR "pervasive developmental disabilit*" OR "global developmental delay*" OR asperger* OR ASD OR HFA OR HFASD OR HF‐ASD OR SCD OR PDD Rett* OR "childhood disintegrative disorder*" OR "triad of impairments" OR "Fragile X" OR PDDNOS OR PDD‐NOS OR PDD/NOS OR savant OR "reactive attachment disorder*" OR AS/HFA OR Kanner* OR aspies OR "childhood schizophrenia" OR "atypical personality development"

### Video‐based interventions

video* OR technology OR mobile OR "video based intervention*" OR "self model*ing" OR "peer model*ing" OR "video feedback" OR imitation OR "video tape*" OR "video model*ing" OR "point‐of view" OR "video prompt" OR "computer*based" OR "in‐vivo model*ing" OR "collaborative tech*" OR "multimedia" OR "applied behaviour* analysis" OR "applied behavior* analysis" OR "functional behaviour* assessment*" OR "functional behavior* assessment*" OR "token econom*" OR "abolishing operation*" OR "ABC checklist" OR "differential reinforce*" OR "abstinence reinforce*" OR "interobserver agreement" OR "schedule* of reinforce*" OR "backward chaining" OR "forward chaining" OR "different* reinforce*" OR "differential reinforce*of alternative behaviour" OR "prompting behavior" OR "prompting behaviour" OR "continuous reinforce*" OR "direct assessment*" OR "delay of reinforce*" OR "contingent escape" OR "functional analys*" OR "continuous reinforcement schedule*" OR "precision teaching" OR "antecedent intervention*" OR "function based intervention*" OR "discrete trail training" OR "extinction burst" OR "motivating operation*" OR "establishing operation*" OR "extinction schedule*" OR "fading prompt*" OR "functional alternative behavior*" OR "transition assessment*" OR "stimulus preference assessment*" OR "positive punishment*" OR "positive reinforce*" OR "negative punishment*" OR "negative reinforce*"OR "early intensive behavi* intervention" OR "discrete trial" OR "intensive behavi* intervention" OR "early behavi* intervention" OR "early applie* behavi* intervention"

### Outcomes

"social behavio*r" OR "social acceptance" OR "social interaction" OR sharing OR approval OR "social change" OR competenc* OR control OR "social desirability" OR "social adjustment" OR mobilit*OR network* OR "social* adapti*" OR interpersonal OR "pro*social" OR "social engagement" OR "social understanding" OR imaginat* OR "social skill*" OR communit* OR vocational* OR domestic OR safety OR academic*

### Terms for children aged 3–11 (will be expanded to capture 11–18‐year olds)

Child* OR "pre school*" OR youth* OR pupil OR Nurser* OR "early childhood education*" OR "primary education" OR Kindergarten OR Elementary OR "Primary class*" OR "Primary school*" OR "reception class* " OR Post‐primary OR "1st year* " OR "First Year* " OR "Junior high" OR "Middle school" OR Age*3 OR "3 year* old*" OR Age*4 OR "4 year* old*" OR Age*5 OR "5 year* old*" OR Age*6 OR "6 year* old*" OR Age*7 OR "7 year* old*" OR Age*8 OR "8 year* old*" OR Age*9 OR "9 year* old*" OR Age*10 OR "10 year* old*" OR Age*11 OR "11 year* old*" OR "Junior infant*" OR "First Class" OR "Second Class" OR "Fourth Class" OR prekindergarten OR "Grade* 1" OR "Grade* 2" OR "Grade* 3" OR "Grade* 4" OR "Grade* 5" OR "Grade* 6 " OR "Grade* one " OR "Grade* three" OR "Grade* four " OR "Grade* five" OR "Grade* six" OR "3rd Grade" OR "4th Grade" OR "6th Grade" OR "First Grade" OR "Second Grade" OR "Third Grade" OR "Fourth Grade" OR "Fifth Grade" OR "Sixth Grade" OR "Group 1" OR "Group 2" OR "Group 5" OR "Group four" OR "First Standard" OR "Second Standard" OR adolescent* OR teen* OR "young adult*"

## References

[cl21171-bib-0001] Additional References

[cl21171-bib-0002] Abrahams, B. S. , & Geschwind, D. H. (2008). Advances in autism genetics: On the threshold of a new neurobiology. Nature Reviews Genetics, 9(5), 341–355. 10.1038/nrg2346 PMC2756414 18414403

[cl21171-bib-0003] Akmanoglu, N. , & Tekin‐Iftar, E. (2011). Teaching children with autism how to respond to the lures of strangers. Autism, 15(2), 205–222. 21339247 10.1177/1362361309352180

[cl21171-bib-0004] Alcantara, P. R. (1994). Effects of videotape instructional package on purchasing skills of children with autism. Exceptional Children, 61(1), 40–55.

[cl21171-bib-0005] American Psychiatric Association (APA) . (2013). Diagnostic and statistical manual of mental disorders (DSM‐5®). American Psychiatric Pub.

[cl21171-bib-0006] Bandura, A. (1969). Social‐learning theory of identificatory processes. In David A. Goslin Handbook of socialization theory and research (213, p. 213–262). Chicago, United States: Rand McNally & Company.

[cl21171-bib-0007] Baron‐Cohen, S. , Jane, A. , & Gillberg, C. (1992). Can autism be detected at 18 months? The needle, the haystack and the CHAT. British Journal of Psychiatry, 161, 839–843. 10.1192/bjp.161.6.839 1483172

[cl21171-bib-0008] Bellini, Scott (2006). The development of social anxiety in adolescents with autism spectrum disorders. Focus on Autism and Other Developmental Disabilities, 21(3), 138–145.

[cl21171-bib-0009] Bellini, S. , & Akullian, J. (2007). A meta‐analysis of video modeling and video self‐modeling interventions for children and adolescents with autism spectrum disorders. Exceptional Children, 73(3), 264–287.

[cl21171-bib-0010] Bereznak, S. , Ayres, K. M. , Mechling, L. C. , & Alexander, J. L. (2012). Video self‐prompting and mobile technology to increase daily living and vocational independence for students with autism spectrum disorders. Journal of Developmental and Physical Disabilities, 24(3), 269–285.

[cl21171-bib-0011] Berthoz, S. , & Hill Elisabeth, L. (2005). The validity of using self‐reports to assess emotion regulation abilities in adults with autism spectrum disorder. European Psychiatry, 20(3), 291–298. 15935431 10.1016/j.eurpsy.2004.06.013

[cl21171-bib-0012] Berument, S. K. , Michael, R. , Catherine, L. , Andrew, P. , & Anthony, B. (1999). Autism screening questionnaire: Diagnostic validity. The British Journal of Psychiatry, 175(5), 444–451. 10789276 10.1192/bjp.175.5.444

[cl21171-bib-0013] Boudreau, J. , & Harvey, M. T. (2013). Increasing recreational initiations for children who have ASD using video self modeling. Education and Treatment of Children, 36(1), 49–60.

[cl21171-bib-0014] Buggey, Tom (2007). A picture is worth: Video self‐modeling applications at school and home. Journal of Positive Behavior Interventions, 9(3), 151–158.

[cl21171-bib-0015] Cannella‐Malone, H. , Sigafoos, J. , O'Reilly, M. , de la Cruz, B. , Chaturi, E. , & Lancioni Giulio, E. (2006). Comparing video prompting to video modeling for teaching daily living skills to six adults with developmental disabilities. Education and Training in Developmental Disabilities, 344–356.

[cl21171-bib-0016] Centre for Reviews and Dissemination, University of York, & Akers Jo . (2009). In J. Akers , R. Aguiar‐Ibáñez & A. Baba‐Akbari (Eds.), Systematic reviews: CRD's guidance for undertaking reviews in health care. Centre for Reviews and Dissemination, University of York. York, UK.

[cl21171-bib-0017] Charlop‐Christy, M. H. , & Daneshvar, S. (2003). Using video modeling to teach perspective taking to children with autism. Journal of Positive Behavior Interventions, 5(1), 12–21.

[cl21171-bib-0018] Chiang, H.‐M. (2008). Expressive communication of children with autism: The use of challenging behaviour. Journal of Intellectual Disability Research, 52(11), 966–972. 18205752 10.1111/j.1365-2788.2008.01042.x

[cl21171-bib-0019] Chung, K.‐M. , Shaye, R. , Matt, M. , Josiah, D. , Todd, M. , & Tassé Marc, J. (2007). Peer‐mediated social skills training program for young children with high‐functioning autism. Research in Developmental Disabilities, 28(4), 423–436. 16901676 10.1016/j.ridd.2006.05.002

[cl21171-bib-0020] Cohen, J. (1960). A coefficient of agreement for nominal scales. Educational and Psychological Measurement, 20(1), 37–46.

[cl21171-bib-0021] Collaboration Campbell . (2014). Campbell systematic reviews: Policies and guidelines. Campbell Systematic Reviews, 1.

[cl21171-bib-0022] Constantino, J. N. , & Todd, R. D. (2000). Genetic structure of reciprocal social behavior. American Journal of Psychiatry, 157(12), 2043–2045. 10.1176/appi.ajp.157.12.2043 11097975

[cl21171-bib-0023] Constantino, J. N. , Davis, S. A. , Todd, R. D. , Schindler, M. K. , Gross, M. M. , Brophy, S. L. , Metzger, L. M. , Shoushtari, C. S. , Splinter, R. , & Reich, W. (2003). Validation of a brief quantitative measure of autistic traits: comparison of the social responsiveness scale with the autism diagnostic interview‐revised. Journal of Autism and Developmental Disorders, 33(4), 427–433. 12959421 10.1023/a:1025014929212

[cl21171-bib-0024] Constantino, J. N. , Davis, S. A. , Todd, R. D. , Schindler, M. K. , Gross, M. M. , Brophy, S. L. , Metzger, L. M. , Shoushtari, C. S. , Splinter, R. , & Reich, W. (2003). Validation of a brief quantitative measure of autistic traits: Comparison of the social responsiveness scale with the autism diagnostic interview‐revised. Journal of Autism and Developmental Disorders, 33(4), 427–433. 12959421 10.1023/a:1025014929212

[cl21171-bib-0025] Constantino, J. N. , & Todd, R. D. (2003). Autistic traits in the general population: A twin study. Archives of General Psychiatry, 60(5), 524–530. 12742874 10.1001/archpsyc.60.5.524

[cl21171-bib-0026] Constantino, J. N. , Gruber Christian, P. , Sandra, D. , Stephanie, H. , Natalie, P. , & Thomas, P. (2004). The factor structure of autistic traits. Journal of Child Psychology and Psychiatry, 45(4), 719–726. 15056304 10.1111/j.1469-7610.2004.00266.x

[cl21171-bib-0027] Constantino, J. N. , & Gruber, C. P. (2012). *Social Responsiveness Scale: SRS‐2*. Western Psychological Services. ​​​​

[cl21171-bib-0028] D'Ateno, P. , Mangiapanello, K. , & Taylor Bridget, A. (2003). Using video modeling to teach complex play sequences to a preschooler with autism. Journal of Positive Behavior Interventions, 5(1), 5–11.

[cl21171-bib-0029] Dahlgren, S. O. , & Christopher, G. (1989). Symptoms in the first two years of life. European Archives of Psychiatry and Neurological Sciences, 238(3), 169–174. 2721535 10.1007/BF00451006

[cl21171-bib-0030] Dapretto, M. , Davies, M. S. , Pfeifer, J. H. , Scott, A. A. , Sigman, M. , Bookheimer, S. Y. , & Iacoboni, M. (2006). Understanding emotions in others: Mirror neuron dysfunction in children with autism spectrum disorders. Nature Neuroscience, 9(1), 28–30. 16327784 10.1038/nn1611 PMC3713227

[cl21171-bib-0031] Dautenhahn, K. , & Werry, I. (2004). Towards interactive robots in autism therapy: Background, motivation and challenges. Pragmatics & Cognition, 12(1), 1–35.

[cl21171-bib-0032] Dawson, G. , & Osterling, J. (1997). Early intervention in autism. In Michael J. Guralnick The effectiveness of early intervention (pp. 307–326). Baltimore, United States: Paul H. Brookes Publishing Co.

[cl21171-bib-0033] Delano, M. E. (2007). Video modeling interventions for individuals with autism. Remedial and Special Education, 28(1), 33–42.

[cl21171-bib-0034] Dixon‐Woods, M. , Bonas, S. , Booth, A. , Jones, D. R. , Miller, T. , Sutton, A. J. , Shaw, R. L. , Smith, J. A. , & Young, B. (2006). How can systematic reviews incorporate qualitative research? A critical perspective. Qualitative research, 6(1), 27–44.

[cl21171-bib-0035] Dunlap, G. , Lee, K. , & Jonathan, W. (2001). ABA and academic instruction. Focus on Autism and Other Developmental Disabilities, 16(2), 129–136.

[cl21171-bib-0036] Duvall, J. A. , Ake, L. , Cantor Rita, M. , Todd Richard, D. , Constantino John, N. , & Geschwind Daniel, H. (2007). A quantitative trait locus analysis of social responsiveness in multiplex autism families. American Journal of Psychiatry, 164(4), 656–662. 10.1176/ajp.2007.164.4.656 17403980

[cl21171-bib-0037] Edwards, L. A. (2014). A meta‐analysis of imitation abilities in individuals with autism spectrum disorders. Autism Research, 7(3), 363–380. 24863681 10.1002/aur.1379

[cl21171-bib-0038] Fleiss, J. L. (1971). Measuring nominal scale agreement among many raters. Psychological Bulletin, 76(5), 378–382.

[cl21171-bib-0039] Fombonne, E. (2005). The changing epidemiology of autism. Journal of Applied Research in Intellectual Disabilities, 18(4), 281–294.

[cl21171-bib-0040] Gast, D. L. , Vincent, W. , Mark, W. , & Farmer Jacqueline, A. (1992). Teaching first‐aid skills to students with moderate handicaps in small group instruction. Education and Treatment of Children, 101–124.

[cl21171-bib-0041] Graetz, J. E. , Mastropieri, M. A. , & Scruggs, T. E. (2006). Show time: Using video self‐modeling to decrease inappropriate behavior. Teaching Exceptional Children, 38(5), 43–48.

[cl21171-bib-0042] Grandin, T. (1996). My experiences with visual thinking sensory problems and communication difficulties. Center for the Study of Autism.

[cl21171-bib-0043] Gresham, F. M. (1986). Conceptual issues in the assessment of social competence in children. In P. S. Strain , M. J. Guralnick & H. M. Walker (Eds.), Children's social behavior (pp. 143–179). Elsevier.

[cl21171-bib-0044] Grynszpan, O. , Weiss, P. L. , Fernando, P.‐D. , & Eynat, G. (2014). Innovative technology‐based interventions for autism spectrum disorders: A meta‐analysis. Autism, 18(4), 346–361. 24092843 10.1177/1362361313476767

[cl21171-bib-0045] Gutstein, S. E. , & Whitney, T. (2002). Asperger syndrome and the development of social competence. Focus on Autism and Other Developmental Disabilities, 17(3), 161–171.

[cl21171-bib-0046] Haring, T. G. , Kennedy, C. H. , Adams, M. J. , & Pitts‐Conway, V. (1987). Teaching generalization of purchasing skills across community settings to autistic youth using videotape modeling. Journal of Applied Behavior Analysis, 20(1), 89–96. 3583966 10.1901/jaba.1987.20-89 PMC1285955

[cl21171-bib-0047] Hedges, L. V. , Elizabeth, T. , & Johnson, M. C. (2010). Robust variance estimation in meta‐regression with dependent effect size estimates. Research Synthesis Methods, 1(1), 39–65. 26056092 10.1002/jrsm.5

[cl21171-bib-0048] Hendricks, D. R. , Smith, M. D. , & Wehman, P. (2009). Teaching youth for success. In P. Wehman , M. D. Smith & C. Schall (Eds.), Autism and the transition to adulthood. Paul H. Brookes Publishing Co.

[cl21171-bib-0049] Higgins, K. , & Boone, R. (1996). Creating individualized computer‐assisted instruction for students with autism using multimedia authoring software. Focus on Autism and Other Developmental Disabilities, 11(2), 69–78.

[cl21171-bib-0050] Higgins, J. P. T. , James, T. , Jacqueline, C. , Miranda, C. , Tianjing, L. , Page, M. , & Welch, V. (2019). Cochrane handbook for systematic reviews of interventions. John Wiley & Sons.

[cl21171-bib-0051] Hine, J. F. , & Wolery, M. (2006). Using point‐of‐view video modeling to teach play to preschoolers with autism. Topics in Early Childhood Special Education, 26(2), 83–93.

[cl21171-bib-0052] Howlin, P. (1997). Prognosis in autism: Do specialist treatments affect long‐term outcome? European Child & Adolescent Psychiatry, 6(2), 55–72. 9257087 10.1007/BF00566668

[cl21171-bib-0053] International Advisory Group for the Revision of ICD‐10 Mental and Behavioural Disorders . (2011). A conceptual framework for the revision of the ICD‐10 classification of mental and behavioural disorders. World Psychiatry, 10(2), 86–92. 21633677 10.1002/j.2051-5545.2011.tb00022.x PMC3104876

[cl21171-bib-0054] Isong, I. A. , Rao, S. R. , Holifield, C. , Iannuzzi, D. , Hanson, E. , Ware, J. , & Nelson, L. P. (2014). Addressing dental fear in children with autism spectrum disorders: A randomized controlled pilot study using electronic screen media. Clinical Pediatrics, 53(3), 230–237. 24391123 10.1177/0009922813517169

[cl21171-bib-0055] Jennes‐Coussens, M. , Joyce, M.‐E. , & Cyndie, K. (2006). The quality of life of young men with Asperger syndrome: A brief report. Autism, 10(4), 403–414. 16908482 10.1177/1362361306064432

[cl21171-bib-0056] Jordan, R. , & Sarah, L. (2011). Developing and using play in the curriculum. In Stuart Powell & Rita Jordan Autism and learning (Classic Edition) (pp. 35–49). Routledge.

[cl21171-bib-0057] Kaale, A. , Smith, L. , & Sponheim, E. (2012). A randomized controlled trial of preschool‐based joint attention intervention for children with autism. Journal of Child Psychology and Psychiatry, 53(1), 97–105. 21883204 10.1111/j.1469-7610.2011.02450.x

[cl21171-bib-0058] Kanner, L. (1943). Autistic disturbances of affective contact. Nervous Child, 2(3), 217–250. 4880460

[cl21171-bib-0059] Keenan, M. , & Nikopoulos, C. (2006). Video modelling and behaviour analysis: A guide for teaching social skills to children with autism. Jessica Kingsley Publishers.

[cl21171-bib-0060] Keenan, C. , Connolly, P. , & Stevenson, C. (2016). PROTOCOL: Universal preschool‐ and school‐based education programmes for reducing ethnic prejudice and promoting respect for diversity among children aged 3‐11: A systematic review and meta‐analysis. Campbell Systematic Reviews, 12(1), 1–45.

[cl21171-bib-0061] Kleeberger, V. , & Mirenda, P. (2010). Teaching generalized imitation skills to a preschooler with autism using video modeling. Journal of Positive Behavior Interventions, 12(2), 116–127.

[cl21171-bib-0062] Koegel, R. L. (1988). *How to teach pivotal behaviors to children with autism: A training manual*.

[cl21171-bib-0063] Kozlowski, A. M. , Matson, J. L. , & Belva, B. C. (2012). Social skills differences between the autism spectrum disorders. Journal of Developmental and Physical Disabilities, 24(2), 125–134.

[cl21171-bib-0064] Laursen, B. , Bukowski, W. M. , Kaisa, A. , & Jari‐Erik, N. (2007). Friendship moderates prospective associations between social isolation and adjustment problems in young children. Child Development, 78(4), 1395–1404. 17650145 10.1111/j.1467-8624.2007.01072.x PMC2754761

[cl21171-bib-0065] LeBlanc, L. A. , Coates, A. M. , Daneshvar, S. , Charlop‐Christy, M. H. , Morris, C. , & Lancaster, B. M. (2003). Using video modeling and reinforcement to teach perspective‐taking skills to children with autism. Journal of Applied Behavior Analysis, 36(2), 253–257. 12858990 10.1901/jaba.2003.36-253 PMC1284438

[cl21171-bib-0066] Lipsey, M. W. , & Wilson, D. B. (2001). Practical meta‐analysis. SAGE publications, Inc.

[cl21171-bib-0067] Machalicek, W. , O'Reilly, M. F. , Natasha, B. , Jeff, S. , & Lancioni, G. E. (2007). A review of interventions to reduce challenging behavior in school settings for students with autism spectrum disorders. Research in Autism Spectrum Disorders, 1(3), 229–246.

[cl21171-bib-0068] Mason, R. A. K. (2012). Meta‐analysis of video based modeling interventions for individuals with disabilities: Procedure, participant, and skill specificity. A&M University.

[cl21171-bib-0069] Mason, R. A. , Davis Heather, S. , Boles, M. B. , & Goodwyn, F. (2013). Efficacy of point‐of‐view video modeling: A meta‐analysis. Remedial and Special Education, 34(6), 333–345.

[cl21171-bib-0070] Mason, R. A. , Davis Heather, S. , Ayres Kevin, M. , Davis John, L. , & Mason Benjamin, A. (2016). Video self‐modeling for individuals with disabilities: A best‐evidence, single case meta‐analysis. Journal of Developmental and Physical Disabilities, 28(4), 623–642.

[cl21171-bib-0071] Matson, J. L. , Dempsey, T. , & Fodstad, J. C. (2009). The effect of autism spectrum disorders on adaptive independent living skills in adults with severe intellectual disability. Research in Developmental Disabilities, 30(6), 1203–1211. 19450950 10.1016/j.ridd.2009.04.001

[cl21171-bib-0072] McCoy, K. , & Hermansen, E. (2007). Video modeling for individuals with autism: A review of model types and effects. Education and Treatment of Children, 30, 183–213.

[cl21171-bib-0073] McCurdy Barry, L. , & Shapiro Edward, S. (1988). Self‐observation and the reduction of inappropriate classroom behavior. Journal of School Psychology, 26(4), 371–378.

[cl21171-bib-0074] Mechling, L. (2005). The effect of instructor‐created video programs to teach students with disabilities: A literature review. Journal of Special Education Technology, 20(2), 25–36.

[cl21171-bib-0075] Moher, D. , Liberati, A. , Tetzlaff, J. , Altman, D. G. , & Grp, P. (2009). Preferred reporting items for systematic reviews and meta‐analyses: the PRISMA statement (Reprinted from *Annals of Internal Medicine*). Physical Therapy, 89, 873–880. 19723669

[cl21171-bib-0076] Mundy, P. , Sigman, M. , & Kasari, C. (1994). Joint attention, developmental level, and symptom presentation in autism. Development and Psychopathology, 6(3), 389–401.

[cl21171-bib-0077] Murphy, O. , Healy, O. , & Leader, G. (2009). Risk factors for challenging behaviors among 157 children with autism spectrum disorder in Ireland. Research in Autism Spectrum Disorders, 3(2), 474–482.

[cl21171-bib-0078] Nijhof, S. L. , Vinkers, C. H. , van Geelen, S. M. , Duijff, S. N. , Achterberg, E. J. M. , Van Der Net, J. , Veltkamp, R. C. , Grootenhuis, M. A. , van de Putte, E. M. , Hillegers, M. H. J. , van der Brug, A. W. , Wierenga, C. J. , Benders, M. J. N. L. , Engels, R. C. M. E. , van der Ent, C. K. , Vanderschuren, L. J. M. J. , & Lesscher, H. M. B. (2018). Healthy play, better coping: The importance of play for the development of children in health and disease. Neuroscience & Biobehavioral Reviews, 95, 421–429. 30273634 10.1016/j.neubiorev.2018.09.024

[cl21171-bib-0079] Nikopoulos, C. K. , & Keenan, M. (2003). Promoting social initiation in children with autism using video modeling. Behavioral Interventions: Theory & Practice in Residential & Community‐Based Clinical Programs, 18(2), 87–108.

[cl21171-bib-0080] Nikopoulos, C. K. , & Keenan, M. (2004). Effects of video modeling on social initiations by children with autism. Journal of Applied Behavior Analysis, 37(1), 93–96. 15154221 10.1901/jaba.2004.37-93 PMC1284483

[cl21171-bib-0081] Norman, J. M. , Collins, B. C. , & Schuster, J. W. (2001). Using an instructional package including video technology to teach self‐help skills to elementary students with mental disabilities. Journal of Special Education Technology, 16(3), 5–18.

[cl21171-bib-0082] Ospina, M. B. , Krebs Seida, J. , Clark, B. , Karkhaneh, M. , Hartling, L. , Vandermeer, B. , Smith, V. , & Tjosvold, L. (2008). Behavioural and developmental interventions for autism spectrum disorder: A clinical systematic review. PLoS One, 3(11), e3755. 19015734 10.1371/journal.pone.0003755 PMC2582449

[cl21171-bib-0083] Popple, B. , Wall, C. , Flink, L. , Powell, K. , Discepolo, K. , Keck, D. , Mademtzi, M. , Volkmar, F. , & Shic, F. (2016). Brief report: Remotely delivered video modeling for improving oral hygiene in children with ASD: A pilot study. Journal of Autism and Developmental Disorders, 46(8), 2791–2796. 27106570 10.1007/s10803-016-2795-4 PMC4939106

[cl21171-bib-0084] Rayner, C. , Carey, D. , & Jeff, S. (2009). Video‐based intervention for individuals with autism: Key questions that remain unanswered. Research in Autism Spectrum Disorders, 3(2), 291–303.

[cl21171-bib-0085] Reichow, B. , Barton, E. E. , Boyd, B. A. , & Hume, K. (2012). Early intensive behavioral intervention (EIBI) for young children with autism spectrum disorders (ASD). Cochrane Database of Systematic Reviews (10). 10.1002/14651858.CD009260.pub2 23076956

[cl21171-bib-0086] Rosen, R. , Weiss, P. L. , Zancanaro, M. , & Gal, E. (2017). Usability of a video modeling computer application for the vocational training of adolescents with autism spectrum disorder. British Journal of Occupational Therapy, 80(4), 208–215.

[cl21171-bib-0087] Rosenberg, M. (2015). Society and the adolescent self‐image. Princeton university press.

[cl21171-bib-0088] Rosnow, R. L. , & Rosenthal, R. (1996). Computing contrasts, effect sizes, and counternulls on other people's published data: General procedures for research consumers. Psychological Methods, 1(4), 331–340.

[cl21171-bib-0089] Rosnow, R. L. , Rosenthal, R. , & Rubin, D. B. (2000). Contrasts and correlations in effect‐size estimation. Psychological Science, 11(6), 446–453. 11202488 10.1111/1467-9280.00287

[cl21171-bib-0090] Russell, G. , Rodgers, L. R. , Ukoumunne, O. C. , & Ford, T. (2014). Prevalence of parent‐reported ASD and ADHD in the UK: findings from the Millennium Cohort Study. Journal of Autism and Developmental Disorders, 44(1), 31–40. 23719853 10.1007/s10803-013-1849-0

[cl21171-bib-0091] Rutter, M. , Andersen‐Wood, L. , Beckett, C. , Bredenkamp, D. , Castle, J. , Groothues, C. , Kreppner, J. , Keaveney, L. , Lord, C. , & O'Connor, T. G. (1999). Quasi‐autistic patterns following severe early global privation. Journal of Child Psychology and Psychiatry, 40(4), 537–549. 10357161

[cl21171-bib-0092] Samson, A. C. , & Hegenloh, M. (2010). Stimulus characteristics affect humor processing in individuals with Asperger syndrome. Journal of Autism and Developmental Disorders, 40(4), 438–447. 19859795 10.1007/s10803-009-0885-2

[cl21171-bib-0093] Sandieson, R. (2006). Pathfinding in the research forest: The pearl harvesting method for effective information retrieval. Education and Training in Developmental Disabilities, 401–409.

[cl21171-bib-0094] Sandieson, R. W. , Kirkpatrick, L. C. , Sandieson, R. M. , & Zimmerman, W. (2010). Harnessing the power of education research databases with the pearl‐harvesting methodological framework for information retrieval. The Journal of Special Education, 44(3), 161–175.

[cl21171-bib-0095] Schopler, E. , Reichler, R. J. , DeVellis, R. F. , & Daly, K. (1980). Toward objective classification of childhood autism: Childhood Autism Rating Scale (CARS). Journal of Autism and Developmental Disorders, 10, 91–103. 6927682 10.1007/BF02408436

[cl21171-bib-0096] Schreibman, L. , Whalen, C. , & Stahmer, A. C. (2000). The use of video priming to reduce disruptive transition behavior in children with autism. Journal of Positive Behavior Interventions, 2(1), 3–11.

[cl21171-bib-0097] Self, T. , Scudder, R. R. , Weheba, G. , & Crumrine, D. (2007). A virtual approach to teaching safety skills to children with autism spectrum disorder. Topics in Language Disorders, 27(3), 242–253.

[cl21171-bib-0098] Shipley‐Benamou, R. , Lutzker, J. R. , & Taubman, M. (2002). Teaching daily living skills to children with autism through instructional video modeling. Journal of Positive Behavior Interventions, 4(3), 166–177.

[cl21171-bib-0099] Siegel, B. , Anders, T. F. , Ciaranello, R. D. , Bienenstock, B. , & Kraemer, H. C. (1986). Empirically derived subclassification of the autistic syndrome. Journal of Autism and Developmental Disorders, 16(3), 275–293. 3558288 10.1007/BF01531660

[cl21171-bib-0100] Sigafoos, J. , Mark, O. R. , & De La Cruz, B. (2007). How to use video modeling and video prompting. Pro‐Ed.

[cl21171-bib-0101] Silverman, L. B. , Bennetto, L. , Campana, E. , & Tanenhaus, M. K. (2010). Speech‐and‐gesture integration in high functioning autism. Cognition, 115(3), 380–393. 20356575 10.1016/j.cognition.2010.01.002

[cl21171-bib-0102] Simpson, R. L. (2001). ABA and students with autism spectrum disorders: Issues and considerations for effective practice. Focus on Autism and Other Developmental Disabilities, 16(2), 68–71.

[cl21171-bib-0103] Stahmer, A. C. (1999). Using pivotal response training to facilitate appropriate play in children with autistic spectrum disorders. Child Language Teaching and Therapy, 15(1), 29–40.

[cl21171-bib-0104] Steinborn, M. , & Knapp, T. J. (1982). Teaching an autistic child pedestrian skills. Journal of Behavior Therapy and Experimental Psychiatry, 13(4), 347–351. 7166609 10.1016/0005-7916(82)90083-0

[cl21171-bib-0105] Sterne, J. A. C. , & Egger, M. (2005). Regression methods to detect publication and other bias in meta‐analysis. In H.R. Rothstein A.J. Sutton & M. Borenstein Publication bias in meta‐analysis: Prevention, assessment and adjustments (pp. 99–110). New York: Wiley.

[cl21171-bib-0106] Tangney, J. P. , Baumeister, R. F. , & Boone Angie, L. (2004). High self‐control predicts good adjustment, less pathology, better grades, and interpersonal success. Journal of Personality, 72(2), 271–324. 15016066 10.1111/j.0022-3506.2004.00263.x

[cl21171-bib-0107] Tantam, D. (2000). Psychological disorder in adolescents and adults with Asperger syndrome. Autism, 4(1), 47–62.

[cl21171-bib-0108] Tetreault, A. S. , & Lerman, D. C. (2010). Teaching social skills to children with autism using point‐of‐view video modeling. Education and Treatment of Children, 33(3), 395–419.

[cl21171-bib-0109] Thelen, M. H. , Fry, R. A. , Fehrenbach, P. A. , & Frautschi, N. M. (1979). Therapeutic videotape and film modeling: a review. Psychological Bulletin, 86(4), 701–720. 482483

[cl21171-bib-0110] Thiemann, K. S. , & Goldstein, H. (2001). Social stories, written text cues, and video feedback: Effects on social communication of children with autism. Journal of Applied Behavior Analysis, 34(4), 425–446. 11800183 10.1901/jaba.2001.34-425 PMC1284338

[cl21171-bib-0111] Travers, J. C. , Kyle, H. , Tom, P. , Randall, B. , Susan, M. , & Richard, T. (2011). Emergent literacy skills of preschool students with autism: A comparison of teacher‐led and computer‐assisted instruction. Education and Training in Autism and Developmental Disabilities, 46, 326–338.

[cl21171-bib-0112] Trimmer, E. , McDonald, S. , & Rushby Jacqueline, A. (2017). Not knowing what I feel: Emotional empathy in autism spectrum disorders. Autism, 21(4), 450–457. 27246093 10.1177/1362361316648520

[cl21171-bib-0113] Van Laarhoven, T. , Winiarski, L. , Blood, E. , & Chan, J. M. (2012). Maintaining vocational skills of individuals with autism and developmental disabilities through video modeling. Education and Training in Autism and Developmental Disabilities, 447–461.

[cl21171-bib-0114] Venham, L. L. , Gaulin‐Kremer, E. , Edward, M. , Dana, B.‐A. , & Jan, C. (1980). Interval rating scales for children's dental anxiety and uncooperative behavior. Pediatric Dentistry, 2(3), 195–202. 6938934

[cl21171-bib-0115] Waddington, H. , Snilstveit, B. , Hombrados, J. , Vojtkova, M. , Phillips, D. , Davies, P. , & White, H. (2014). Farmer field schools for improving farming practices and farmer outcomes: A systematic review. Campbell Systematic Reviews, 10(1), i–335.

[cl21171-bib-0116] Waddock, S. A. , & Graves, S. B. (1997). The corporate social performance–financial performance link. Strategic Management Journal, 18(4), 303–319.

[cl21171-bib-0117] Walker, H. M. , Kate, K. , Bruce, S. , Annemieke, G. , Severson Herbert, H. , & Feil Edward, G. (1998). First step to success: An early intervention approach for preventing school antisocial behavior. Journal of Emotional and Behavioral Disorders, 6(2), 66–80.

[cl21171-bib-0118] Wang, S.‐Y. , Ying, C. , & Parrila, R. (2011). Examining the effectiveness of peer‐mediated and video‐modeling social skills interventions for children with autism spectrum disorders: A meta‐analysis in single‐case research using HLM. Research in Autism Spectrum Disorders, 5(1), 562–569.

[cl21171-bib-0119] Weiss, M. J. , & Harris, S. L. (2001). Teaching social skills to people with autism. Behavior Modification, 25(5), 785–802. 11573340 10.1177/0145445501255007

[cl21171-bib-0120] Wolff, S. (2004). The history of autism. European Child & Adolescent Psychiatry, 13(4), 201–208. 15365889 10.1007/s00787-004-0363-5

